# On the Changes Which Take Place in the Lungs after Division of the Pneumogastric Nerves

**Published:** 1849-06

**Authors:** 


### RECORD OF MEDICAL SCIENCE.

### ANATOMY AND PHYSIOLOGY.

*On the Changes which take place in the Lungs after Division of the Pneumogastric Nerves.* By Dr. Schiff.—It is well known that after section of the vagus nerves in the neck of an animal, death frequently takes place at an interval of a few days. In these cases the lungs are found to have undergone alterations, characterised by congestion, and the effusion of a large quantity of frothy sanguinolent serum into the bronchi; which lesions have been ascribed by authors to the paralysis of the glottis consequent on the section of its nerve, (the recurrent,) which induces respiratory obstruction, either directly, or by permitting the passage of food and other matters into the trachea. Dr. Schiff has performed a variety of experiments which disprove these ideas. By cutting in some animals the recurrents, and in others the pulmonary branches of the vagus, he has convinced himself that the section of the latter causes congestion, with tumefaction of the bronchial mucous membrane; while that of the former only produces narrowing and paralysis of the glottis, without any pulmonary changes. The lesions of the lungs are likewise unaffected by the performance of tracheotomy, and by the section of the œsophagus in the neck (in dogs.) He therefore concludes, that the state of the lungs is dependent on the integrity of the pulmonary portion only of the nerve. Section of the nerve on one side produced a slighter amount of pulmonary lesion, but never confined to the lung of one side ; a circumstance which M. Schiff accounts for by considering the anastomoses in the pulmonary plexus of nerves.—*Archiv. für Physiologische Heilkunde.*

[Dr. J. Reid considers the congestion and bronchial effusion as a secondary effect of the diminished frequency of respiration in animals in which the vagi are divided.—(Ed. Med. and Surg. Journal, April 1839.) This idea agrees perfectly with the results of the above experiments; and is, we doubt not, quite correct.]—*Lond. Month. Jour.*

